# Navigating the social world with neck dystonia: An Interpretative Phenomenological Analysis

**DOI:** 10.1177/13591053241286131

**Published:** 2024-10-12

**Authors:** Melanie Maxwell-Scott, Fiadhnait O’Keeffe, Fiona JR Eccles

**Affiliations:** 1 Lancaster University, UK; 2 St Vincent’s University Hospital Dublin, Ireland; 3 University College Dublin, Ireland

**Keywords:** adults, cervical dystonia, interpretative phenomenological analysis, neck dystonia, qualitative, social support, social world, spasmodic torticollis, stigma

## Abstract

Neck dystonia is a neurological condition, characterised by involuntary movements of the neck muscles, causing twisted head positions and often pain and head tremor. Ten participants with neck dystonia were interviewed and the data was analysed using an Interpretative Phenomenological Analysis approach. Three themes were constructed: (1) dismissed by others for having an unfamiliar condition; (2) negotiating a new social identity; and (3) managing the stigma of a visible condition. It is proposed that psychological support could benefit people with neck dystonia who experience difficulties arising from identity management and stigma. Systemic interventions, such as education campaigns, are also proposed to help address stigmatising attitudes.

## Introduction

Dystonia refers to a group of neurological conditions characterised by sustained or intermittent muscle contractions and spasms (Albanese et al., 2018). Neck dystonia, also known as cervical dystonia or spasmodic torticollis, is the most common form of focal dystonia (affecting specific body parts) with an estimated prevalence of 9.95 per 100,000 (Medina et al., 2022). Involuntary movement of the neck muscles leads to twisted head postures, and can be accompanied by pain and tremor (Albanese et al., 2023). Treatment for neck dystonia is focused on reducing motor symptoms and often involves regular injections of botulinum toxin into the affected muscles (Albanese et al., 2018). Onset of neck dystonia is usually between the ages of 30 and 50 years old (Defazio et al., 2013) meaning many people experience diagnosis when they are working and/or caring for family members (Skogseid et al., 2005). Women are affected 1.7 times more often than men (Defazio et al., 2013). Neck dystonia is a chronic condition, although approximately 11.8% of people may experience a temporary remission (Mainka et al., 2019).

In addition to motor symptoms, people with neck dystonia also commonly experience a range of psychological difficulties including poor sleep, sensory issues, anxiety and depression (Kuyper et al., 2011; Smit et al., 2017; Stamelou et al., 2012). These difficulties have been shown to have a greater impact on health-related quality of life than motor symptoms (Klingelhoefer et al., 2021; Ndukwe et al., 2020). This has led to debates as to whether psychological distress is a primary feature of dystonia (Ndukwe et al., 2020), or a consequence of living with this chronic condition (Comella and Bhatia, 2015; Lewis et al., 2008).

One such consequence of living with a chronic condition can be the experience of stigma. Goffman (1963) described a stigmatised individual or group as possessing features which differ from a social norm, either due to physical differences, ‘character flaws’ or identification with a particular group on the grounds of, for example, race or religion. One study found 51% of participants with neck dystonia reported the feeling of stigmatisation and this was particularly prevalent for those under 60 years (Klingelhoefer et al., 2021). However, there is limited and conflicting evidence as to how stigma affects the wellbeing and quality of life of people with neck dystonia (Basurović et al., 2012; Ben-Shlomo et al., 2002).

Furthermore, stigma may be only part of the experience of interacting in a social world and quantitative studies are limited in that it is difficult to capture individual experiences of how society and identity interact with the physical body. Qualitative studies overcome this limitation by exploring issues in depth. One such study which examined experiences of living with dystonia found that the stigma of an unfamiliar and visible condition had a negative impact on participants’ sense of self-identity (Morgan et al., 2021). However, the Morgan et al. (2021) study explored the broad experience of living with dystonia, rather than focusing on specific aspects of that experience, and included many different forms of dystonia, so the experiences of living with specific symptoms were not elucidated. Consequently, the present study aims to build on the limited qualitative and quantitative literature on the psychological effects of neck dystonia by focusing on the social implications of living with this condition. An improved understanding of the experience and impact of neck dystonia will help psychologists and other health professionals when supporting members of this population. The research question was: what are the social experiences of people with neck dystonia?

## Method

### Design

The study followed an Interpretative Phenomenological Analysis (IPA) approach, as outlined by Smith et al. (2021). IPA is considered a suitable design as it is in-depth and can capture the interaction of social and psychological factors (Eatough and Smith, 2017). Ethics approval for the project was granted by the Lancaster University Faculty of Health and Medicine Research Ethics Committee.

### Participants

Individuals were eligible to take part if they (a) were adults between the ages of 35 and 65; (b) self-reported having a diagnosis of idiopathic neck dystonia for at least a year; (c) were English speaking; (d) were able to participate in an interview either online or by telephone. The age range was selected to capture people mid-life who were likely to be (or have been) in employment, in long-term relationships and possibly with caring responsibilities. People who were diagnosed less than a year previously were excluded as they were considered to be in the initial stages of understanding the condition. People were invited to attend regardless of severity of symptoms or whether they were undergoing treatment.

A total of 10 participants took part in the study aged 38–64 (mean = 54.7 years; Table 1). Seven were female and three were male. All participants were British, and currently living in the UK. Four participants were working, with various employment statuses, and five were retired. To protect anonymity, each participant was asked to pick a pseudonym.

**Table 1.** Participant demographics.

Pseudonym	Gender	Age	Years since diagnosis	Employment status	Relationship status	Children
Aggie	F	58	7.5	Retired	Married	2
Bridget	F	63	20	Retired	Married	2
Christian	M	44	15	Employed	Married	2
James	M	64	14	Retired	Married	2
Lucy	F	38	1	Employed	Married	3
Philip	M	59	13	Retired	Married	2
Rachel	F	46	7	Employed	Partner	0
Sarah	F	59	1.1	Unemployed	Married	3
Shadow	F	55	6	Employed	Married	2
Susan	F	61	30	Retired	Married	2

### Procedure

Dystonia UK, the largest charity supporting people with dystonia in the UK, shared a recruitment poster with its members. Interested individuals made contact with the researcher and were sent a participant information sheet and consent information. At the beginning of each interview the consent form was read aloud to the participant, and they were audio-recorded giving verbal consent.

The interview topic guide was informed by previous research relating to social experiences of people with other movement conditions (Barker et al., 2014; Desborough et al., 2020; Theed et al., 2017) and developed in consultation with an expert by experience. Questions fell into four broad areas: background and clinical information; social relationships; context to social relationships, including home, travelling on public transport, online world; and social identity, including motivation to socialise and attitudes to other people. Example questions included: Has dystonia changed your views of what makes a good friend? Does your partner understand how the dystonia impacts on you? How did dystonia impact your relationship with work colleagues? However, rather than sticking rigidly to the topic guide, cues were taken from participants as to where they wanted to take the interview. This meant the interview covered topics that were most important to the participant (Smith et al., 2021). Follow-up prompts such as ‘can you say more about that?’ or ‘can you explain what that meant to you?’ encouraged participants to expand on their responses. Five participants chose to take part via MS Teams, and five by telephone. Interviews ranged in length from 41 to 113 minutes (mean = 63 minutes).

For analysis, each interview was transcribed verbatim and then each transcript analysed in turn to enable an idiographic approach following six steps (Smith et al., 2021). Step 1 involved reading and re-reading the transcript to allow for active engagement with the data and to enter ‘the participant’s world’ (Smith et al., 2021: 80). The second step involved going through the transcript and recording anything of interest and the third step involved constructing experiential statements (Smith et al., 2021), which relate directly to the participant’s experience and how they are making sense of their experience. The fourth step involved looking for common themes across the experiential statements to develop ‘personal experiential themes’ (PETs) and the fifth step involved naming the PETs and consolidating them in a table. Step 6 involved repeating steps 1–5 for each participant and finally for looking for similarities across the PETs in order to create a set of ‘group experiential themes’ (GETs). The PETs for each participant and their contribution to the GETs can be seen in Supplemental Table 1 (online).

Analysis was conducted by the first author and all stages of the data collection and analysis process were reviewed by at least one of the two other authors to improve coherence and impact (Yardley, 2000). A reflexive journal and regular discussion with the second and third authors were used by the lead author to raise awareness of underlying perspectives and biases that may influence the process (Creswell, 2012).

## Findings

The process of analysis yielded three GETs: (1) dismissed by others for having an unfamiliar condition; (2) negotiating a new social identity; and (3) managing the stigma of a visible condition. All participants contributed to each of the GETs.

### GET 1: Dismissed by others for having an unfamiliar condition

The first GET related to how participants experienced other people’s reactions to them having a rare and poorly understood condition. All 10 participants reported the onset of dystonia symptoms as a worrying period marked by the uncomfortable sensations of the head being twisted and, in some cases, shaken. The path to diagnosis involved multiple appointments with different healthcare professionals, often resulting in participants having to return time and again to have their symptoms taken seriously. This seemed particularly hurtful for participants due to an expectation that health professionals should have understood the significance of dystonia. Susan’s experience was one of belittling: ‘*The GP looked at it and said oh you’ve got a wry neck and the general response of all the medics was that they laughed, they literally laughed*’. Bridget was told by the GP her symptoms were the result of her self-consciousness of being tall, whereas Bridget was ‘*proud of being tall*’.

Following diagnosis, participants were left reeling in shock at their new reality of living with a chronic condition for which there are treatments, but no cure. Both Aggie and Shadow described how the ‘*rug was pulled out from under my feet*’ (Aggie), suggesting how suddenly life had been turned upside down. For all participants, in varying ways, neck dystonia had an all-encompassing and often limiting impact on life. The condition meant participants lost jobs, income, the freedom to drive and use public transport, the ability to enjoy hobbies, exercise and play musical instruments. As Aggie explained with a level of exasperation: ‘*I’ve had to give up work. I can’t drive. Everything. I’m uncomfortable or in pain 24 hours a day*’. All participants described a feeling of exhaustion as though they were in constant battle with their bodies: ‘*I am fighting it all the time, all day … it is really tiring … I compare it to pushing two opposing magnets against each other … that’s what it’s like in your neck all the time*’. (Christian).

In stark contrast to the considerable imposition neck dystonia placed on the lives of participants, they all described being met by others’ dismissive attitudes and minimising comments. Rachel experienced such attitudes from her employer: ‘*I mean she as good as said that she didn’t believe the diagnosis*’. For Shadow the lack of understanding came from her mother: ‘*She [mother] doesn’t understand, and she’s not interested in the fact that I can’t do most things*’. Aggie experienced a lack of awareness from her friends as to how difficult it was to live with physical symptoms of neck dystonia, as she explained: ‘*A few people have said to me, “oh, yeah I know exactly what you’re going through because I clicked my neck two months ago and it was bad.”* [But she wanted to respond] *“You haven’t got the foggiest idea what you’re talking about.”*’

However, there was a recognition by some participants that they would need to be more explicit in order that others could understand. For example, Christian noted how his friends assumed dystonia was similar to a pulled muscle because he had not spelt out how difficult it was for him: ‘*The fact that my friends, and it was quite hurtful, never really took the time to understand … and I think this is more my fault because of the way that I powered through*’. Bridget noted how once she had posted a video online about how hard things had been for her, she received several supportive messages: ‘*she* [friend] *phoned me up. She said, “oh I’m crying.” I said, “why?” and she said, “oh Bridget, I didn’t realise”*’. This suggests other people were not all being purposefully ignorant, rather they just needed help to understand.

A connection with others built on shared understanding of the condition was an important coping strategy for some participants, as James noted when joining a dystonia support group: ‘*So immediately there was somebody there I could speak to who had been on exactly the same journey … that was a massive, massive help*’. Another important coping strategy seemed to be the participants’ need to address this general lack of understanding regarding neck dystonia. All 10 participants spoke of joining support groups, raising awareness through social media and/or contributing to research.

### GET 2: Negotiating a new social identity

This GET related to how the disabling nature of neck dystonia led to changes in the social groups that participants belonged to. Given all participants were of working age, the impact dystonia had on ability to work featured prominently in their accounts of living with the condition. For example, Sarah had to stop working due to a head tremor that meant she could no longer focus on computer screens. This was significant as work had been an important part of Sarah’s identity, acting as a constant thread throughout her life: ‘*I have always worked*’. Sarah’s future identity as someone who worked was also taken away from her by neck dystonia: ‘*I think I envisaged going on a bit longer than the age I am, at work. And now I can’t do that. It is upsetting, yeah. Not to have the choice really*’. (Sarah)

Having to take ill-health retirement from her job meant Shadow lost income and needed to claim state benefits. Work symbolised Shadow’s place in the world, it represented her intelligence, her abilities and her contribution to society. Not being able to work meant Shadow belonged to a ‘*different part of society to the one I was used to being in*’. Even while in work, some participants felt that they had lost status due to their health condition. For instance, although Philip found his employer to be supportive following his dystonia diagnosis, he described being moved to a new department to ‘*gather dust*’, reflecting a sense of no longer feeling important or relevant to the operation. Philip experienced a conflict of, on the one hand, wanting to prove to colleagues he could still do his job, and on the other hand, having to accept his colleagues’ assistance: ‘*I’ve got quite a bit of pride I don’t like being helped with things, but on the other hand if it helps to get the job done*’.

For some participants, neck dystonia marked the advent of new, and often positive, ways of working. Three participants explained how they now worked remotely. Technology had allowed them to manage their jobs around physical symptoms, for example, being able to rest when needed, and to avoid customers and clients noticing their symptoms. Managing to overcome difficult periods of work history was described with a sense of pride by some participants. For example, Rachel left her job due to ill-health after her dystonia diagnosis and took the opportunity to start her own business, as she reflected: ‘*I do wonder that we have to be kind of pushed - don’t we sometimes? - to make the change that we want to change*’.

Changing work patterns also meant a readjustment of social relationships for some participants. Shadow described how she found herself outside her social ‘*bubbles*’, which had been linked to her employment status: ‘*They focus on themselves. They’re like little bubbles … And once you’re out of that bubble, everybody else carries on*’. The idea of bubbles suggest a stratified social environment, in which people belong to different groups governed by certain rules of membership. Once she left her work bubble, Shadow’s colleagues no longer stayed in touch with her. The transition from being able bodied to having a physical disability enabled Shadow to see her social world for what it was, which she describes with a sense of sadness and remorse that she had not been able to see this before: ‘*I was really only there as a work colleague … And that I find with hindsight a little bit unsettling. Really, I was only there for what I did. I wasn’t there for me*’.

The physical difficulties experienced by participants also led to lessons being learnt about themselves. For instance, Lucy described a new clarity in understanding of what is important to her: ‘*I would try to prioritise the people - all the things that I really want to do - and forget the rest*’. Some participants reflected on how living with neck dystonia had changed the way they related to other people, for example, becoming more sensitive to others’ distress, as Bridget explained: ‘*I’m nicer, nicer with people. And I think about their, their problems and if they’re feeling uncomfortable … I’d bend over backwards to try and make people feel comfortable*’.

### GET 3: Managing the stigma of a visible condition

The third GET related to how neck dystonia is a condition which is visible to others. This had an impact on how participants saw themselves and how they imagined others saw them. For instance, participants repeatedly used the words ‘*weird*’ and ‘*strange*’ to describe their symptoms, reflecting how they had internalised a particular physical presentation as ‘normal’ and now saw them themselves as deviating from that, and felt that others would perceive this unnatural deviation too. For example, Susan described her experience of working in a public facing job: ‘*They maybe think you look a bit weird. They’re thinking, “why is this weird person talking to me?” You know? “What’s the matter with them?”*’ Several participants reported a sense of being stared at, and even judged or assessed by others: ‘*If you go shopping or something you notice people’s eyes sort of travelling up your head up to your eyes. I know what women must feel like now with men’s eyes. It’s a very strange feeling*’. (Philip)

Being looked at by strangers, without being spoken to, left an unnerving gap in the social interaction, which felt one-sided with power weighted towards the stranger without the visible difference. Participants struggled with not knowing what people were thinking about them. Shadow suspected that people were misrepresenting her as someone with less intelligence: ‘*… they talk slower. And they talk louder. There’s nothing wrong with my hearing*’. Other people’s stares were also internalised by participants and experienced as shame or self-consciousness. Bridget described how she changed from being a confident young person to one who feared being around other people. She blamed this fear on the ‘*unwelcome friend*’ that is neck dystonia. It manifested as a constant voice in her head which commentated on how she looked to other people: ‘*I’ve got this person inside me that even now … it’s always there from the moment I wake up to the moment I go to sleep*’. Lucy reported a similar experience of self-consciousness: ‘*I’m trying to have a conversation with somebody, but in the back of my mind I’m thinking about how bad does this look*’. Self-consciousness acted as a vicious cycle for some participants, as being stared at made the symptoms worse, which then made the self-consciousness worse, as Christian explained: ‘*Anxious, anxiety, tiredness, all these different things. It can [exacerbate] the dystonia, so my shaking goes into overdrive, and I feel very self-conscious then*’.

The existence of stigmatised attitudes meant Sarah felt blamed for her neck dystonia. This led to her feeling embarrassed to tell people about her condition and even to ask for medical help: ‘*I know it isn’t my fault, but I think you do feel embarrassed and keep on saying there’s something wrong. No, I don’t really talk about it*’. However, Rachel took an alternative position and considered how the visibility of dystonia had the benefit of showing to people that you do need help, and helped other people to understand how difficult dystonia is: ‘*Because if people can’t see that you’re struggling with whatever or that you have a condition, then you are not treated any differently*’.

To manage the impact of stigma, all 10 participants described strategies they had developed which had varying degrees of positive and negative consequences. Some strategies were designed to conceal the physical symptoms of neck dystonia, for example, using gestures to disguise a head tremor, sitting at certain angles to disguise head position and leaning the head on a hand while sitting down. These strategies often came at the expense of causing pain. Some participants reduced the time they spent amongst strangers, thereby increasing the risk of social isolation: ‘*It [neck dystonia] would stop me from creating any new friendships … because I wouldn’t put myself in a situation where I’d meet anybody*’ (Aggie). Bridget described how she had used alcohol to quieten her self-conscious thoughts, to stop worrying about other people looking at her, and to some extent, to lessen her physical symptoms. Although she did not use this strategy anymore at the point of interview, it had had a significant impact on her daily activity: ‘*Every single time I went out socially from about 21 onwards, I had to have a drink every time … I have made such a fool of myself in family situations*’. Mitigating stigma using cognitive strategies was another option for participants. For example, some tried to ignore the staring, explaining the condition to people, or accepted that stigma was an inevitable part of ‘*human nature*’ (Christian). Philip used humour to both explain to people that he had the visible implants from a brain operation and to lessen his and others’ awkwardness: ‘*… when I used to say I’m part cyborg - that’s usually a good ice breaker*’.

## Discussion

This research aimed to understand the ways in which neck dystonia impacts on individuals’ social interactions. The GETs highlighted how participants experienced the dismissive attitudes of others given the unfamiliarity of the condition, how their social identity evolved following the changes brought on by physical symptoms and how they experienced and coped with the stigma of a visible difference. The challenge in negotiating the social world is interrelated with the physical difficulties, pain and exhaustion that neck dystonia brings.

In the first GET, participants discussed their confusion and shock at the onset of neck dystonia symptoms. This resonates with the findings from the Morgan et al. (2021) paper which described participants as ‘*struggling to escape the darkness*’ (p. 946) as they sought to find answers for their condition. In contrast to the enormity of this experience, participants in the present study were confronted by other people’s lack of awareness and understanding. Words or actions that are intended to exclude and invalidate people has been termed the ‘disavowal of disability’ (Hughes, 2007: 681). Hughes (2007) theorises that other people’s fears of physical difference and social exclusion are projected onto those who show such vulnerability. Consequently, a ‘hierarchy of existence’ is created, with people who are non-disabled at the top (Hughes, 2007: 681). This can lead to oppression of those at the bottom by those at the top through psycho-emotional disablism, which impacts individuals’ sense of worth and self-esteem (Thomas, 2007). By being made to feel that their condition was not important, this suggests that participants were experiencing this type of oppression.

The second GET highlighted how a change in social networks and roles impacted on participants’ sense of self-identity. Unexpected life changes can affect our relationships to other people and how other people see us, which can disrupt the continuity of identity (Haslam et al., 2021). Some of the participants seemed to accept the shifting social groups brought on by the onset of neck dystonia, whereas others experienced more distress. This difference could relate to the Social Identity Model of Identity Change (SIMIC; Jetten et al., 2009; Jetten and Pachana, 2012), which posits that a person’s capacity to successfully negotiate life changes depends on how previously developed social identities provide support to facilitate the establishment of new identities (e.g. see Barker et al., 2014). Those participants in the present study who found support from family and groups of old friends, unrelated to work, may have been able to more easily adapt to their new identity than those who did not have access to that support.

Distress associated with changes in social identity could also be related to stigma. For instance, some participants reported their discomfort with becoming known as someone who does not work, who is cared for by relatives or needs to claim ill-health benefits. Such consequences are often related to health conditions, and neck dystonia is not a condition that is easily hidden from others. A large body of research exists which links stigma to visible health conditions (e.g. Maffoni et al, 2017; Mayor et al., 2022; Smith et al., 2015). The third GET showed how stigma can then be internalised as shame, embarrassment or self-consciousness. This suggests participants were endorsing negative beliefs and feelings associated with their stigmatised condition and applying them to the self (Link and Phelan, 2001). Internalised stigma is thought to be more disruptive to an individual’s life than enacted stigma, that is, actual discrimination by others (Scambler and Hopkins, 1986). Therefore, it makes sense that participants would make considerable efforts to mitigate the effects of this stigma. Similar findings were reported in studies of people with Tourette’s syndrome, who disguise and misattribute their visible symptoms by seeking solitude and disguising tics as intentional movements (Buckser, 2008). Concealment of symptoms by people with chronic health conditions has been found to deter people from seeking social support and medical treatment (Earnshaw and Quinn, 2012). Thus potentially contributing to difficulties navigating new social identities.

Overall, the findings provide support for the theoretical concept of disablism (Thomas, 2007), in that people with neck dystonia experience both structural oppression and psycho-emotional disablism. Social support enabled participants to renegotiate their identities as suggested by the SIMIC, and helped to buffer them from the stigma of having a visible, unfamiliar condition. Quantitative studies show that psychosocial factors such as stigma and self-esteem have a significant impact on quality of life and mood (Ben-Shlomo et al., 2002), and that illness perceptions are related to distress in people with neck dystonia (O’Connor et al., 2022). Findings from the present study add depth to this evidence by describing the complex ways these interrelated factors are experienced.

### Limitations

The homogenous sample of participants, as is required by IPA, means the results are not generalisable to younger or older people, and those from different cultures. Only nationality was recorded, not ethnicity, which means some British ethnic populations were not represented. Future research involving non-white and non-Western participants would be beneficial. This is especially the case given cultural differences in how stigma is manifested and expressed in relation to chronic health conditions and visible differences (Abdullah and Brown, 2011). Stigma is a social construction which depends on a power differential between the stigmatised and the stigmatisers (Link and Phelan, 2001). There are other social constructions which create power and privilege in societies, for example, race, gender and age (Rosenthal, 2016). The research is therefore limited by viewing stigma solely through a visible difference perspective. Future research should seek to understand how different social identities intersect when living with neck dystonia.

Participants were recruited through Dystonia UK, which could lead to selection bias. For instance, people involved with a charity may be more interested in campaigning because they find such support to be helpful themselves, they may be more likely to have greater formal education and hunt out such opportunities, or they may have experienced more negativity from society than others with neck dystonia and wish to talk about their experiences. Alternatively, the participants may have been those who were less stigmatised and therefore happy to be interviewed, as very embarrassed and self-conscious people may not come forward to volunteer for research projects. It was notable that all the participants were in a relationship with a partner, husband or wife, and that this relationship was a significant contributor to social support. Further studies could also obtain partner and family perspectives to understand in more detail the nature of social support and its impact on wellbeing.

### Clinical implications

Given the nature of this small scale qualitative study, it would be premature to make generalised clinical recommendations. Nonetheless, the current study offers insights into experiences for which clinical intervention could be helpful. Typical treatment for neck dystonia is aimed at managing the motor symptoms (Albanese et al., 2018), however it is clear from the findings from the current research that psychosocial features of the condition interact with motor symptoms and contribute to distress. Given the experiences of feeling dismissed (GET 1) and self-stigma (GET 3) the provision of psychoeducation may act as a suitable first-line intervention to increase positive attitudes to the condition which may in turn reduce distress (O’Connor et al., 2022). Similarly, group interventions could foster a sense of understanding from others to help overcome the feelings of invalidation (GET 1). A 3-day, mindfulness-based, group residential programme for participants with different forms of dystonia found the group dynamic legitimated their condition leading to a reduction in distress (Sandhu et al., 2016).

GET 2 highlighted the negotiation of social roles that was required and other therapeutic approaches could be considered to help understand and adapt underlying beliefs about an individual’s identity and the condition (see Austin et al., 2021; Konstantinou et al., 2023). For example, cognitive-behavioural therapy could be a suitable approach for addressing negative body concept (Lewis et al., 2008) and for helping people to deal with the consequences of self-stigma, such as feelings of embarrassment (Corrigan and Calabrese, 2005), as highlighted in GET 3. Acceptance and Commitment Therapy has been recommended to help support cognitive flexibility in people with multiple sclerosis, which may act as a buffer between stigma and wellbeing (Valvano et al., 2016). Compassion focused therapy has also been found to be an effective approach to treating shame associated with chronic illness (Carvalho et al., 2022). Both these approaches could help address the stigma indicated in GET 3. Future research could explore the effectiveness of these interventions with a dystonia population.

However, it would not be appropriate simply to offer individualised treatment for psychological distress caused by stigma without also addressing its origin. Given participants’ experiences in GET 1 and GET 3 of being misunderstood, dismissed and belittled by others, systemic interventions are also required to reduce stigmatising attitudes, for example, educational campaigns aimed at increasing public awareness of the condition (see Patalay et al., 2017). Given the experience the participants reported during the diagnosis process (GET 1) educational campaigns should also be targeted at GPs and healthcare professionals. This could include recommendations for health professionals to draw on the resources of people’s social relationships (Haslam et al., 2005; Jelinek and Hassed, 2009), for example, by aiming to have a better understanding of their patient’s social identities and meaningful activities. The media, academics and charities also have a role in disseminating information, with the help of healthcare professionals, to paint a more accurate picture of conditions that, although, life-restricting can be treated and adapted to.

## Conclusion

This study explored participants’ experiences of navigating the social world with neck dystonia. Participants spoke about the dismissive attitudes they experienced from other people regarding their diagnoses, how their social identities have changed, and how they internalised and coped with the impact of stigma from a chronic and visible condition. These findings highlight the important interaction of psychosocial experiences with physical symptoms. While Morgan et al.’s (2021) findings covered several types of dystonia, the focus here was on neck dystonia, and shows how difficult it could be to negotiate the social world with a very visible physical difference, which is poorly recognised and understood. The current paper provides more in depth exploration of the different ways social identities changed and also more detail about internalised stigma and distress as a response to the social context. Both individual psychological support and targeted approaches to reduce stigma could help improve the social experience of people with neck dystonia.

## Supplemental Material

sj-docx-1-hpq-10.1177_13591053241286131 – Supplemental material for Navigating the social world with neck dystonia: An interpretative phenomenological analysis Supplemental material, sj-docx-1-hpq-10.1177_13591053241286131 for Navigating the social world with neck dystonia: An interpretative phenomenological analysis by Melanie Maxwell-Scott, Fiadhnait O’Keeffe and Fiona JR Eccles in Journal of Health Psychology
