# Supplementary material for: Navigating the social world with neck dystonia: An Interpretative Phenomenological Analysis
Source: J Health Psychol. 2024 Oct 12;30(10):2719–30. doi: 10.1177/13591053241286131 (PMC12381391; doi:10.1177/13591053241286131)
Supplement: sj-docx-1-hpq-10.1177_13591053241286131 – Supplemental material for Navigating the social world with neck dystonia: An interpretative phenomenological analysis [file sj-docx-1-hpq-10.1177_13591053241286131.docx]

**Supplementary table 1:** **Contributing personal experiential themes (PETs) to each group experiential theme (GETs), with example quotes.**

| **Participant** | **Theme 1 - Dismissed by others for having an unfamiliar condition** | **Theme 2 - Negotiating a new social identity** | **Theme 3 - Managing the stigma of a visible condition** |
| --- | --- | --- | --- |
| Aggie | Journey of dystonia – from shock to grief to acceptance  *“Although I'm much more accepting of the situation now, much more accepting. It's still my whole life revolves around this wretched condition.”* | Renegotiate identity  *“I've always been, you know, very particular home maker and having to rely on my husband to help me cook the meals, do so many things he has to help. with showering to wash my hair and dry my hair and so all the things that you just so much take for granted. Just vanished.”*  Costs and benefits of social relationships have changed  *“I've got a very, very good set of friends, quite a lot. But the acquaintances are just probably they haven't bothered, and neither have I.”* | Visible condition exposes her truth to strangers  *“I still don't really go out that much. Mainly because it's uncomfortable. But also because I don't really want to expose myself. To other people.”*  Pretend everything is normal at home  *“I I feel I'm safe and I'm secure at home. I know my own surroundings and I can almost pretend that everything is normal when I'm at home.”* |
| Bridget | Misunderstood and not heard  *“My dad died when I was eleven, about four years before that and I it was nothing to do with that whatsoever.*  *But they [school nurse and teacher] were thinking it was that, you know, and, um, so I put up with it for a while and I didn't tell my mom because my mum was busy.”* | Conflict of identity  *“I'm nicer, nicer with people. And I think about their, their problems and if they're feeling uncomfortable … I'd bend over backwards to try and make people feel comfortable.”* | Stigma – a vicious cycle  *“Every single time I went out socially from about 21 onwards, I had to have a drink every time…I feel I don't need to have a drink before I go out, but it's taken all this time and I have, I have made such a fool of myself in family situations”.*  Dystonia as the unwelcome friend  *“I've got this person inside me that even now … it's always there from the moment I wake up to the moment I go to sleep”.* |
| Christian | Lack of awareness and understanding from others  *“I actually had to tell my doctor that I had cervical dystonia. And at the time my doctor said, ‘no, you haven't’”.* | Dystonia identity combined with other identities  *“I've got a bit of a mad sense of humour anyway, so it’s easier for me to deal with. And don't get me wrong,*  *there are days where I'll get up and I'm really exhausted and I'm really having a bad time.”*  Fitting world around dystonia vs dystonia to fit around world  *“Working from home - I can set myself in really strange positions. I probably couldn't in the office.”* | Feeling self-conscious from visible condition  *“Anxious, anxiety, tiredness, all these different things. It can [exacerbate] the dystonia, so my shaking goes into overdrive, and I feel very self-conscious then”.*  Stigma of “it’s all in your head”  *“A lot of people in the dystonia community are being told that it's in your head and that it's just a psychological thing. It doesn't really help us out when we turn around and say things like tiredness and things like that are affecting it.”* |
| James | Dismissed by others / dismissing self  *“Felt a bit of a fraud going there when you see people who had brain injuries and car accidents and things and I went there with a slightly bent head, but they [hospital] were superb.”* | Fear of burdening others with needs  *“We try not to burden them* [adult children] *with stuff but they would do anything, and they have, helped out in different ways.”* | Self-consciousness as uncertainty  *“The fact that you look a complete weirdo when your head is, you know, craning in these strange directions… what are they thinking about me?”* |
| Lucy | Unfamiliarity of dystonia is barrier to understanding  *“I haven’t really talked to [husband’s] side of the family. Just seems to be like it's a big thing for me, but it's not really that big a deal… it sounds like a bit of a whinge if I bring up ‘ohh, by the*  *way’.* | Shaping of social life to fit dystonia  *“I've got three kids, so your social life is never that brilliant is it when you've got kids? But definitely do that less now [go out to restaurants]. You’re thinking about…how much discomfort I'm gonna be in, sat in the same bit for ages.”* | Dystonia means a life of self-consciousness.  *“When I'm going about my day, I've kind of learned to disguise it a bit so I'm never still.”* |
| Philip | Regaining control over medical treatment  *“Back in the early 2000s the detail wasn’t too good so I didn’t have a lot to refer to. I had a few printed leaflets off the neurologist but yes, not a lot.”* | Loss of role/ identity through needing help  *“I’ve got quite a bit of pride I don’t like being helped with things, but on the other hand if it helps to get the job done”.* | Reclaiming power over what people think about his visible difference  *“We’d go away for these away-days, and you’d have to introduce yourself with something unusual about yourself – when I used to say I’m part cyborg - that’s usually a good ice breaker.”* |
| Rachel | Power balance with doctor and patient  *“They [doctors] just sort* *of said ‘oh it's probably just* *stress’ and anyway they didn't* *give me anything for it and it* *was a bit of a waste of time.”*  Support means being believed  *“I mean she [employer] as good as said that she didn't* *believe the diagnosis”.* | Insider or outsider of dystonia community  *“It's not something that any of my online clients know anything about or my my private clients now know about because they just never needed to. It would only have been something I would be discussed if I were symptomatic. But because it's so obvious, you don't really have a choice in that regard, you know?”* | Visibility is disclosure to others  *“You know, the fact people can* *see or can't see, it really* *shouldn't be a big deal. But of course it is. Because if people* *can't see that you're struggling* *with whatever or that you have a* *condition, then you are not* *treated any differently, you* *know? And, and it's not about* *needing to be treated* *differently. It's about needing* *to be seen, I think, for people, isn't it?”* |
| Sarah | Learning to accept  *“So people don't know what it is and I've never heard of it. And when you say cervical dystonia, I think they think it's women's problems…It's embarrassing.”* | Limitations – reducing her social world  *“It's just everything if I have visitors in, we have people in for coffee last week and it's looking from one person to another, it sets it off. I Start to feel really queasy. I've had enough after now, it just puts me off the socialising.”* | Embarrassment about what dystonia means about her  *“It feels like always moaning and I feel like that when I contact the doctors too. I know it isn't my fault, but I think you do feel embarrassed and keep on saying there's something wrong. No, I don't really talk about it”.* |
| Shadow | Dystonia – strange and unknown  *“I mean even my mum, we haven't spoken for three or four years now. Because I'm. I'm not the same, you know, and I'm, I'm. So even though you know she's family, it's not. She doesn't understand and she's not interested in the fact that I can't do most things. She just wants to carry on, you know, it's fine. I'll just go, ‘OK, go ahead. I can't do it’. So there's been no contact there for years.”* | Identity as conceptual, economic and social phenomenon  *“And it just, it was just a disaster, you know, because financially and all the rest of it. I have no money. Where was it? No income, you know, nothing. And that kind of puts you in a different. You're in a different part of society to the one I was used to being in.”*  People step up or fall by the wayside  *“I was really only there as a work colleague … And that I find with hindsight a little bit unsettling. Really, I was only there for what I did. I wasn't there for me”.* | Stigma – into the matrix  *“And it made, it's made me think … when I was normal, I suppose, for want of a better word. Did I do the same thing? If there were people in wheelchairs and things, do you do the same thing to them?* *Without even thinking about it. And I was put into that world that I thought.* ***‘****Yeah, people do really speak to disabled people like this’”.* |
| Susan | An individual dystonia story – from violent beginning to power of experience  *“The GP looked at it and said oh you’ve got a wry neck and the general response of all the medics was that they laughed, they literally laughed.”* | Struggle over identity  *“So I found that this gave me a real empathy for people with physical things like cerebral palsy and spasticity because I could understand to a small extent how physically draining and whatever so it helped me to support them in a manner that I hope worked for them well. I think he did, because, you know, they always seem to like me.”* | Stigma – damaged identity  *“They maybe think you look a bit weird. They're thinking, ‘why is this weird person talking to me?’ You know? ‘What's the matter with them?’”.* |
